# Europa’s ice thickness and subsurface structure characterized by the Juno microwave radiometer

**DOI:** 10.1038/s41550-025-02718-0

**Published:** 2025-12-17

**Authors:** S. M. Levin, Z. Zhang, S. J. Bolton, S. Brown, A. I. Ermakov, J. Feng, K. Hand, S. Misra, M. Siegler, D. Stevenson, W. McKinnon, R. Akiba

**Affiliations:** 1 Jet Propulsion Laboratory, California Institute of Technology, Pasadena, CA USA; 2 California Institute of Technology, Pasadena, CA USA; 3 Southwest Research Institute, San Antonio, TX USA; 4 Stanford University, Stanford, CA USA; 5 Planetary Science Institute, Tucson, AZ USA; 6 University of Hawaii, Manoa, HI USA; 7 Washington University in St Louis, St Louis, MO USA; 8 University of California Santa Cruz, Santa Cruz, CA USA

**Keywords:** Rings and moons, Cryospheric science

## Abstract

Jupiter’s moon Europa is thought to harbour a saltwater ocean beneath a variously disrupted ice shell, and it is, thus, one of the highest priority astrobiology targets in the Solar System. Estimates of the ice-shell thickness range from 3 km to over 30 km, and observations by the Galileo spacecraft indicated widespread regions of ice disruption (chaotic terrain) leading to speculation that the ice shell may contain subsurface cracks, faults, pores or bubbles. If persistent, subsurface cracks could provide pathways for habitability by facilitating the transport of oxygen and nutrients between the surface and the ocean. Here we report on observations of Europa’s subsurface ice shell obtained by the Juno microwave radiometer in 2022. For the idealized case of pure water ice, the data are consistent with the existence of a thermally conductive ice shell with a thickness of 29 ± 10 km and with the presence of cracks, pores or other scatterers extending to depths of hundreds of metres below the surface with a characteristic size smaller than a few centimetres in radius. An ice-shell salinity of 15 mg kg^−1^, as indicated by models based on terrestrial marine ice, would reduce our estimate of the thickness of the ice shell by about 5 km, substantially less than our 10 km uncertainty. The low volume fraction, small size and shallow depth of the scatterers indicate that the fracture interfaces observed at Europa’s surface are alone unlikely to be capable of carrying nutrients between the surface and the ocean.

## Abstract (web summary)

Microwave observations of Jupiter’s moon Europa reveal subsurface properties of the ice shell covering a purported global ocean. The shell is 29 ± 10 km thick, with fractures or pores a few centimetres across that extend hundreds of metres deep.

## Main

The Juno spacecraft is spin stabilized with a spin period of 2 rpm. It has been in polar orbit around Jupiter since 2016^1^. On 29 September 2022, the spacecraft flew past Europa at an altitude of 360 km. Europa has been a high priority target for planetary scientists for more than 40 years. Whether Europa is habitable has been debated for decades^2,3^. Interest in habitability substantially increased when measurements by the Galileo spacecraft indicated the presence of an electrically conductive (salty) water ocean beneath the ice crust and fractures in the surface ice^4,5^. During the Juno fly-by of Europa, Juno’s microwave radiometer (MWR), which was designed to observe Jupiter’s deep atmosphere^6–8^, provided spatially resolved measurements of the brightness temperatures at various depths of Europa’s ice-shell crust.

**Fig. 1:**
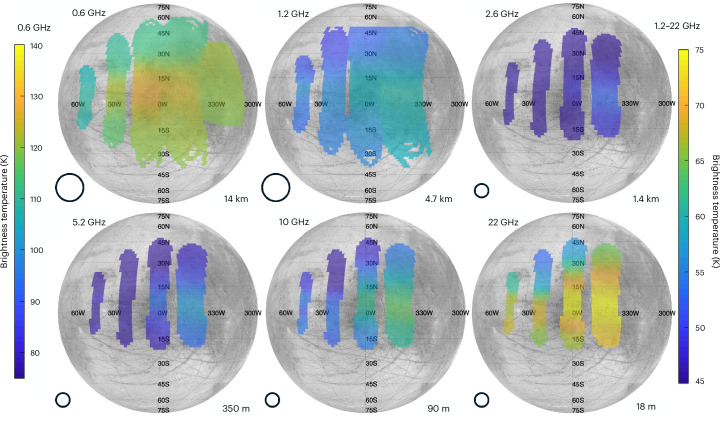
MWR data for each frequency channel, superimposed on a map of Europa. The 0.6-GHz data are much brighter due to reflected synchrotron emission and are, therefore, shown with a different colour scale. The terminator was at approximately 10° W longitude, with the right-hand side in sunlight for these maps. The approximate sounding depth (50% contribution for pure, solid ice) is shown in the lower right of each map, and a typical beam size is shown in the lower left of each map.

MWR is a six-channel set of radiometers operating at 0.6 GHz, 1.2 GHz, 2.6 GHz, 5.2 GHz, 10 GHz and 22 GHz. At Europa, MWR provided a map of thermal emissions emanating from the Europan ice shell at depths ranging from several metres at 22 GHz to several kilometres at 0.6 GHz. These observations of ice temperature versus depth include evidence of frequency-dependent scattering in the ice, and they provide constraints on the thickness of the ice shell along with the size and depth of sources of the observed reflections. Here we present an analysis of the MWR observations of Europa. The dataset comprises measurements made when the boresight emission angles were <60° and excludes data contaminated by non-Europan sources (the antenna pattern was at least 90% on Europa). The dataset includes 129 measurements in each of the six MWR channels, obtained from four consecutive spins of the spacecraft. Figure 1 shows the six MWR maps obtained, covering latitudes ~10° S to ~30° N and longitudes 60° W to 40° E. MWR measured the antenna temperature *T*_A_ with better than 2% absolute accuracy, and the stability and noise yield better than 1 K precision for differences between measurements at the same frequency^6^.

Infrared observations indicate that the region observed of Europa’s surface has temperatures that range from about 90 K to 110 K, with a further diurnal variation of about 20 K (refs. ^9,10^). Although Europa’s surface is predominantly composed of water ice, there is evidence of salts and other non-icy materials^11^, perhaps including sulfur compounds and CO_2_ ice and perhaps organic molecules or rocky materials. Surface morphology and magnetic field measurements indicate the presence of a subsurface ocean^4,5,12–14^. Before Juno, estimates of the ice shell were not well constrained by observation, ranging from a possible ice thickness of ~3 km to ~45 km (refs. ^5,13,15–17^). The presence of a water ocean beneath the ice shell provides a constraint on the vertical temperature gradient (the ocean–ice interface must be ~270 K). A convective ice layer may exist between the ocean and the conductive ice shell^18^. Measurements of the vertical temperature gradient can be used to estimate the thickness of the conductive part of the ice shell, with a possible convective layer adding to the total thickness. An ice regolith layer, with a depth between a few metres and a kilometre, has been hypothesized to contain fractures and voids as large as 75 cm in diameter^11,19–22^. The thickness of the ice shell and the nature of the regolith constrain possible communication between the surface and the ocean, impacting theories of whether Europa is habitable^23,24^.

## Results

All six MWR antennas were positioned to look out perpendicular to the spin axis, and they collected multiple 100 ms samples of the microwave brightness. Each of these samples was calibrated and converted to an on-disk brightness temperature *T*_B_ for each frequency, using the procedure described by Brown et al.^25^. Figure 1 shows the resulting four swaths for each channel, superimposed on six maps of Europa. Spacecraft motion relative to Europa carried the swaths westwards, and the spacecraft spin plane was approximately perpendicular to Europa’s equator, so the swaths are roughly along lines of constant longitude. The five highest frequencies are co-boresighted. The lowest-frequency antenna, 0.6 GHz, is mounted on a different part of the spacecraft and observes approximately 20 s later (thus, it did not observe exactly the same terrain). As a result, the swaths corresponding to 0.6 GHz are displaced in longitude relative to the other observations, and our analysis takes this into account. The data cover a range of emission angles over various terrains, with some observed correlation between emission angle, terrain type and optical albedo. The two main types of terrain on Europa are ridged plains (generally older and brighter) and chaos (highly disrupted and generally younger and darker). On the sub-Jovian hemisphere shown in Fig. 1, the eastern half observed by Juno is dominated by chaos. Synchrotron emission from Jupiter’s radiation belts and from the Galaxy is reflected by Europa, which affected the 0.6-GHz observations and (to a lesser extent) the 1.2-GHz observations.

The range of physical ice temperatures and the various contribution of reflected emission due to both viewing geometry and reflective properties of the ice result in variations in *T*_B_ within the 129-point dataset for each channel. This lateral variation in *T*_B_ is a precise differential measurement, with an uncertainty of less than 0.5 K. Thus, the discrepancies are dominated by modelling assumptions, and measurement error can be neglected. A similar fly-by of Ganymede in 2021 revealed a reflective structure within the ice shell and constrained subsurface temperatures. The data indicated a relation between surface optical albedo and microwave reflectivity and showed that the reflective ice surface was rough on centimetre scales^25,26^.

In the absence of reflection, the brightness temperature, *T*_thermal_, due to thermal emission from the ice is the attenuated integral along the line of sight of the product of ice temperature and opacity. The opacity at frequencies between 0.6 GHz and 22 GHz determines the depth that MWR sounds into the ice. The opacity of pure water ice is a function of both frequency and temperature, with reduced opacity at lower frequencies and at lower temperatures^27^, so the sounding depth at each frequency depends on the temperature profile of the ice. In pure ice at ~100 K with no reflections, most of the emission received by the 0.6-GHz channel would come from deeper than 20 km. A single reflection with reflectivity *R* at the surface of the ice removes some of this thermal emission and adds reflected emission from the sky and results in an observed brightness temperature, *T*_B_, given by:

1 $${T}_{{\rm{B}}}=(1-R){T}_{{\rm{thermal}}}+(R){T}_{{\rm{sky}}}.$$

The sky brightness *T*_sky_ depends on viewing geometry as well as frequency. Subsurface discontinuities, such as cracks, voids or, for example, salt inclusions in the ice, also cause reflections. For each frequency channel, reflectors that are much smaller than the wavelength have very little effect, and reflectors whose dimensions are close to the observing wavelength or larger act as scatterers that spread the reflected emission over a range of directions. When there are several subsurface reflectors, equation (1) is modified to account for complexities, such as the power absorbed and emitted by the ice above each reflector, several bounces and so on (Methods).

**Fig. 2:**
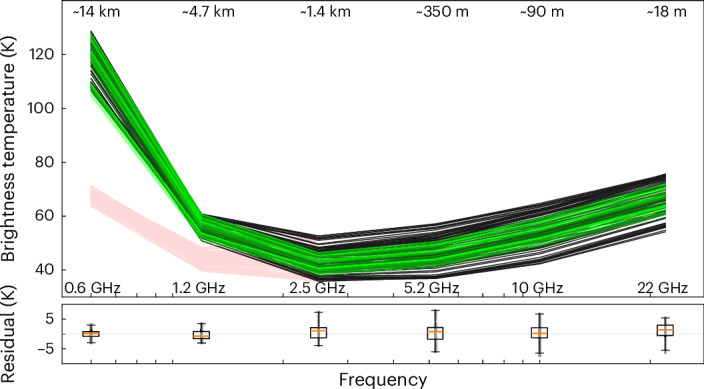
Microwave data and modelled spectra from Europa. The modelled ice shell is laterally uniform and 29 km thick, with reflective pores as described in the text. Data are shown in black, and the model is shown in light green. Pink spectra represent data after subtracting the modelled effects of reflected synchrotron emission. The depth shown at the top is the approximate depth in pure, solid, ice for which 50% of the contribution to each channel comes from above and 50% comes from below. Residuals for the 129 measurements (data minus model) are shown at the bottom, with boxes indicating the median, first and third quartiles, and 1.5 times the interquartile range. See Methods for further details of the model.

All 129 observed spectra are shown in Fig. 2. We note three salient features: (1) *T*_B_ decreases steadily from 22 GHz to 5.2 GHz, corresponding to increasing depth. The liquid ocean beneath the ice layer has a temperature near 273 K, with the surface ice temperature near 100 K, so the physical ice temperature must increase with depth, in contrast to *T*_B_. The observed spectral slope, therefore, indicates the presence of subsurface reflectors that extend to at least the depth seen by the 5.2-GHz channel, because the deeper 5.2-GHz and 10-GHz channels see more reflection from the cold sky than the shallower 22-GHz channel. (2) The spectrum is nearly flat from 1.2 GHz to 5.2 GHz and shows only a modest rise at 0.6 GHz (after subtracting the contribution from reflected synchrotron emission), indicating either that the ice shell is substantially thicker than the depth probed by the lowest frequencies or that reflectors nearly balance out the increase in ice temperature with depth. (3) Much less spread (lateral variation) is observed in *T*_B_ at 1.2 GHz and 0.6 GHz than at higher frequencies. If the source of high-frequency-only lateral variation is reflectors, they are smaller than 25 cm (wavelength of 1.2 GHz) because otherwise the lateral variation would continue to be observed at the lower frequencies as well. Another possibility is that the ice temperature varies laterally at shallow depths and becomes more homogeneous at depths observed with the lower frequencies. This implies a relatively thin ice shell. The ice temperature near the surface varies with albedo and latitude due to solar heating. At depths approaching the ice–liquid interface, we expect these lateral variations to fade away. However, the lateral variation at 0.6 GHz is like the lateral variation at 1.2 GHz, and for a thin shell, the deeper sounding at 0.6 GHz should result in a greatly reduced variation in ice temperature compared with at 1.2 GHz. Thus, a qualitative look at the spectra indicates an ice shell substantially thicker than the sounding depth at 1.2 GHz, along with the presence of reflectors too small to affect the 0.6-GHz and 1.2-GHz channels.

To investigate the subsurface ice structure, the thickness of the ice and the source of reflection, we use a radiative transfer model that incorporates conductive and convective transport. The model allows for surface reflection and volume scattering from subsurface voids (Methods). We further assume: (1) the surface reflectivity and subsurface scatterers do not vary laterally, (2) the scatterers are discontinuities in the ice (for example, pores, voids or cracks), (3) the scattering source has a power-law size distribution and exponentially decreases with depth and (4) the diurnal layer is thin compared with the sounding depth of all channels. The diurnal layer is estimated to be less than 1 m deep^28^, consistent with the lack of a diurnal signal at the highest frequency recorded by MWR. Note that any discontinuity in the dielectric will cause scattering and absorption, regardless of whether the source is a vacuum, liquid or salt pocket. We treat the scatterers as vacuum pores, but allowing for the presence of liquid water or precipitated salt yields very similar conclusions. We vary the power-law index, scale height, volume fraction of the voids, surface reflection and the thickness of the conductive ice shell, and we fit the data with a Markov chain Monte Carlo (MCMC) algorithm^29^. The model shown in Fig. 2 is our best fit to the data, with conductive ice-shell thickness 29 km, negligible surface reflectivity, volume fraction of scatterers 0.045, scale height of scatterers 219 m and power-law index for the scatterer size distribution −3.96. All five of these parameters are constrained not to vary with latitude and longitude. The only properties that vary laterally are the ice temperature, which is driven by albedo and latitude, and the illumination by synchrotron emission, which is dictated by the observing geometry. Note that this laterally uniform model reproduces the average spectrum as well as the spread at 0.6 GHz and 1.2 GHz but does not explain the spread in the data at 2.6 GHz and above. The spread in the model at 0.6 GHz is substantially affected by reflected synchrotron emission, which comes from a fixed direction and, therefore, varies with viewing geometry, even for a model in which the reflectivity is laterally uniform. For all the other channels, the spread in the model is dominated by the effect of optical albedo and latitude-dependent insolation on ice temperature.

**Fig. 3:**
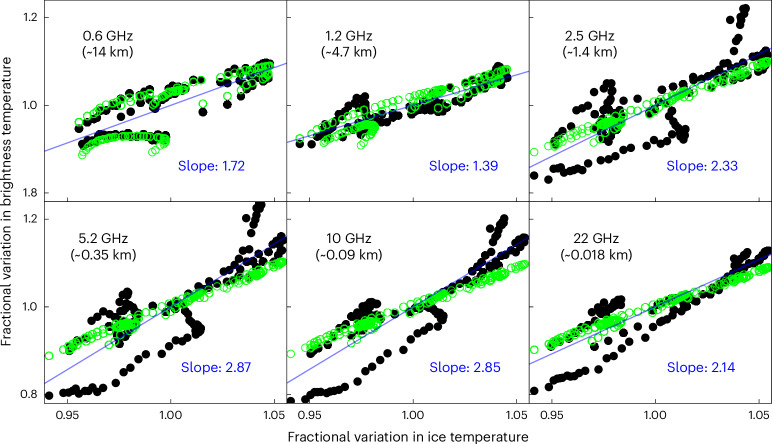
Fractional variation in brightness temperatures versus fractional variation in predicted ice temperature just below the diurnal layer. Observations are shown in black, and a laterally uniform model (the same as in Fig. 2) is shown in light green. A linear fit to the slope of the data is shown in blue.

Figure 3 shows the normalized lateral variation in *T*_B_ versus ice temperature, for both observations and the model, with each divided by its global average to show the fractional variation. Both the thermal emission and the microwave opacity of the ice are functions of ice temperature, causing both the sounding depth of each channel (and, hence, the amount of reflection seen) and the thermal emission to vary, with slopes greater than 1. As noted above, the observed variation in *T*_B_ could be caused by a variation of either the physical ice temperature or reflections. However, the lateral variation observed at 0.6 GHz and at 1.2 GHz, as shown in Fig. 3, matches the modelled variation in ice temperature for a 29-km ice shell, and the excess variation at 2.5 GHz and above does not correlate with ice temperature. Further, the excess lateral variation is clearly correlated across the high-frequency channels, with an amplitude that increases with depth across the shallower channels. This indicates that the excess variation at higher frequencies is largely due to a lateral variation in subsurface reflectors that are too small or too shallow to have much effect on the 0.6-GHz and 1.2-GHz channels and that the ice shell is substantially thicker than the depth sounded at 0.6 GHz.

**Fig. 4:**
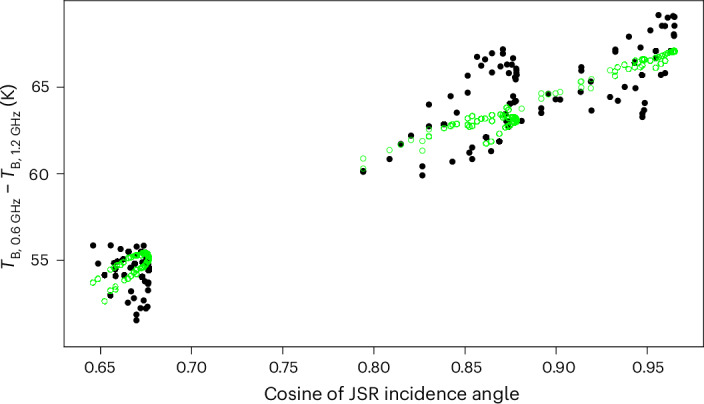
Brightness temperature gradient between 0.6 GHz and 1.2 GHz plotted against the cosine of the incidence angle for synchrotron emission. Data are shown as black filled circles. The model, shown as green open circles, is the same as in Figs. 2 and 3.

The vertical temperature gradient, measured by the difference between the 0.6-GHz and 1.2-GHz channels, constrains the thickness of the conductive layer of the ice shell. A convective layer, if present, would increase the total ice-shell thickness and very slightly decrease our estimate of the conductive layer thickness. The relation between *T*_B_ and physical ice temperature is affected by reflection, but reflected synchrotron emission from the Jovian radiation belts varies with incidence angle and, so, provides a useful constraint on the reflectivity. We assume that the lateral variation in the reflectivity of the ice at 0.6 GHz and 1.2 GHz is not correlated with the illumination by Jupiter’s synchrotron emission. The lateral variation in synchrotron emission constrains the amount of reflection, which in turn must balance the ice temperature gradient to match the data, thus determining the thickness of the ice shell. Figure 4 shows the difference in the brightness temperatures of the 0.6-GHz and 1.2-GHz channels as a function of the synchrotron illumination. Using measurement noise and calibration uncertainty alone, the MCMC results in an average conductive ice-shell thickness of 29 km with a formal uncertainty of 0.2 km. We take into account the extra modelling uncertainty in two ways. Applying the remaining residual in each channel as an estimate of the extra independent uncertainty in each measurement, we add these in quadrature with the measurement uncertainty and run the MCMC again, treating the combined uncertainty as 1.3 K, 1.9 K, 3.0 K, 3.5 K, 3.4 K and 3.2 K for 0.6 GHz to 22 GHz, respectively. This results in the model parameters used in Figs. 2–4, as described above, with average conductive ice-shell thickness 29 km and formal uncertainty ±1 km. An unmodelled systematic lateral variation in the ice properties that conflate with the Jovian synchrotron incidence angle would affect this result. We take the difference between the model and the data shown in Fig. 4 as an estimate of the potential effect of unmodelled lateral variation. The standard deviation of the residuals of *T*_B, 0.6 GHz_ − *T*_B, 1.2 GHz_ is 1.7 K. If an unmodelled lateral variation resulted in a 1.7-K change in *T*_B, 0.6 GHz_ − *T*_B, 1.2 GHz_ that was highly correlated with the effect of synchrotron incidence angle, our estimate of the average conductive ice-shell thickness would change by 9.3 km. Adding this uncertainty in quadrature with the formal uncertainty, we take the average conductive ice-shell thickness of the region measured by MWR to be 29 ± 10 km.

## Discussion

Our analysis of MWR data at Europa shows a conductive ice shell that is 29 ± 10 km deep, assuming pure water ice and no convective ice layer. Our estimate of the conductive ice-shell thickness depends crucially on the thermal gradient implied by measured differences between the 0.6-GHz and 1.2-GHz channels. Impurities in the ice, such as salts or other materials, would increase the microwave opacity, potentially reducing the depth sounded by all channels and, therefore, reducing the difference in depth between the 0.6-GHz and 1.2-GHz channels, with a roughly proportional effect on our estimate of the ice-shell thickness. To determine the importance of this phenomenon, we use effective medium theory to estimate the effect of salinity in the form of inclusions and alternatively estimate the effect of salt dissolved in the ice lattice by adjusting the imaginary part of the dielectric constant^30^ to account for the conductivity contribution of Cl (ref. ^30^). Marine ice with a salinity of 60 μM Cl^−^ (~2.3 mg kg^−1^), as suggested by Blankenship et al.^13^, would have very little effect on our analysis. An ice-shell salinity of 15 mg kg^−1^, as estimated by Steinbrugge et al.^31^, if global, would reduce the sounding depth at 0.6 GHz by about 17% if dissolved in the ice lattice or by about 4% if present in the form of inclusions. Higher levels of salinity, which have been suggested^32^, along with a temperature dependence of their effect on opacity, could have a more substantial impact, although there are relevant hydrated sulfate salts that have little effect on microwave attenuation^33^. To affect the lowest frequencies and decrease the estimate of the conductive ice-shell thickness, impurities would need to be widespread enough to affect the bulk of the MWR data and deep enough to substantially affect the contribution function of the 0.6-GHz channel. The required depth depends on the amount and nature of the impurities. The modelling discussed here, which assumes pure ice, predicts a 50% contribution to the 0.6-GHz channel at 14 km. Impurities that result in a different dependence on ice temperature would also alter the match between the data and the model by changing the modelled slope shown in Fig. 3. Further analysis of this effect may be able to constrain the degree of impurities and further constrain the ice-shell properties.

Our estimate for Europa’s ice shell is towards the thicker end of the range of estimates in the literature but a physically plausible one^17^. The heat flow in our model, which corresponds to a conductive 19–39-km-thick shell, ranges from 15 mW m^−2^ to 35 mW m^−2^. This heat flow is in excess of what is expected for a chondritic radiogenic contribution alone (~10 mW m^−2^) and is consistent with estimates that include tidal heating in Europa’s ice shell^34^. Note that this is an estimate for Europa at the present time. Geophysical models of structures and processes often derive substantially higher heat flows^35^, but these, generally, strictly apply to the formation times of the structures in question, and the thickness of Europa’s shell has probably changed substantially over the ~100-Myr history of its present surface^35^. Models of tidal dissipation also predict substantial variations in shell thickness as a function of latitude and longitude^34^, which our MCMC model does not address. No isostatic topographic evidence has confirmed this predicted variation^35^, however, and it has been argued that oceanic heat transport could essentially eliminate such shell thickness variations on Europa^36^.

We also find that the regolith includes a distribution of scatterers that falls off as the fourth power of pore size and decreases with a scale height of ~220 m. We treat these scatterers as pores but do not rule out cracks or salt inclusions, which would yield similar results. If the ice is impure enough to be substantially more opaque than pure ice, then the scatterers must have a shallower vertical distribution. The lateral variation in the scatterers extends at least a few metres deep but is not seen in the deepest channels. Because of their low volume fraction, shallow depth relative to the depth of the ocean and small size, the pores, voids or fractures implied by our results would probably not, on their own, be a route to supply nutrients to the ocean or to provide ocean-to-surface communication. Our data do not rule out other sources associated with active regions or impacts on Europa or the possibility that the smallest pores, which are undetectable by the deepest sounding frequencies of MWR, do not fall off exponentially but instead continue much deeper. We note that our results are limited to the terrain observed, and further mapping of Europa’s surface by radiometry or radar may reveal regions where the ice shell is thinner or thicker or contains unobserved variations in the regolith.

## Methods

### Calibration and data processing

The raw MWR measurements were converted to antenna temperatures *T*_A_ and calibrated as described by Janssen et al.^6^ and Brown et al.^25^. Observations of the sky acquired during the fly-by were used to remove interchannel biases in *T*_A_ and to flatten the spectral calibration. Biases of −0.13 K, 3.7 K, 4.9 K, 0.6 K, 2.8 K and 0.75 K were subtracted from *T*_A_, respectively, for the 0.6–22-GHz channels. The resulting uncertainty in the absolute brightness temperature was estimated to be <1 K across all channels for the Europa brightness temperature range after this process. To correct for the fraction of the antenna gain pattern that falls off Europa, we applied the same technique as was used for Ganymede data^25^. As was done for Ganymede, we rejected observations with an emission angle larger than 60° or with more than 10% of the antenna power falling off the limb of Europa. The relative precisions among the individual measurements are 0.5 K, 0.4 K, 0.3 K, 0.2 K, 0.2 K and 0.15 K for the 0.6–22-GHz channels, respectively^25^.

### Jovian synchrotron radiation and Galactic radio emission

Because Europa is partially reflective in the MWR frequency range, the antenna temperature received by MWR in each channel includes a component due to reflected Jovian synchrotron radiation (JSR) from Jupiter’s radiation belts and reflected radio emission from the Galaxy. Each of these sources has a highly non-thermal spectrum, with the largest contribution to the 0.6-GHz channel. The contribution at 1.2 GHz is reduced by about a factor of 4, and the corrections at 2.5 GHz and above are successively smaller and rapidly become negligible. The JSR was essentially constant for the duration of our Europa observations but can vary by as much as 25% on longer timescales. To estimate the JSR brightness, we used an approach based on what was done with Ganymede data^26^, simplified to take advantage of the fact that we have direct MWR observations of synchrotron emission during the Europa fly-by. Because of the orientation of the Juno spin plane relative to Jupiter, MWR directly observed approximately half of the synchrotron emission in each 30-s spin of the Juno spacecraft during the Europa fly-by. We used a model of synchrotron emission^37,38^ to adjust for viewing geometry, scaling the modelled map of synchrotron emission to match the direct MWR observations. Galactic radiation (GR) is static and was directly measured during the cruise phase of the mission, preceding Jupiter orbital insertion. The baseline sky level (above the cosmic microwave background), composed of emissions from the Galaxy and unresolved radio sources, was established at 6.6 K, 0.9 K and 0.1 K at frequencies of 0.6 GHz, 1.2 GHz and 2.5 GHz, respectively^39^, with minimal contributions assumed at 5 GHz and above. Because the JSR was measured directly during each spin, the uncertainty in the JSR brightness is less than 1%, and the uncertainty in the effect of JSR is dominated by the uncertainty in the reflectivity of the ice shell.

### Radiative transfer modelling

To explore the thermal and scattering characteristics of Europa, we developed a simple model. This model incorporates estimates of Europan ice-shell viscosity and its surface heat flux, and it allowed us to assess the thickness of both the conductive and convective ice shells as well as the physical temperature gradient within the shell. As depicted in Supplementary Fig. 1, our model incorporates surface reflection and volume scattering in the subsurface. Volume scattering is assumed to be caused by voids in the medium of solid ice. As the scatterers approach the scale of MWR wavelengths, volume scattering becomes frequency-dependent. Any intrinsic thermal emissions from the ice undergo scattering and absorption in the ice as they propagate through the scatterers, with subsequent reflection at the surface layer. Conversely, extrinsic sources, such as JSR and GR, are partially reflected by the reflective layer of the surface and by scatterers beneath the surface.

As illustrated in Supplementary Fig. 1, upwelling intrinsic thermal emission from the ice shell (ray 1) is scattered within the volume-scattering layer before finally reaching the ice–vacuum interface. At the surface, part of the emission is transmitted through the interface and exits the ice layer and part of it is scattered back into the volume-scattering layer. Downwelling thermal emission (ray 2), if not absorbed by the ice shell, might reach the ice–ocean interface and be partially scattered upwards. Extrinsic JSR or GR (ray 3) is partially scattered by the reflecting surface layer and partially enters the ice shell. Once inside the ice shell, this radiation could be scattered by the volume-scattering layer back to the vacuum. If the ice shell is very thin (≤10 km), the JSR and GR could reach the ice–ocean interface and be scattered upwards (ray 4).

The ice shell is modelled as a water-ice medium embedded with scatterers whose volume fraction exponentially decays with depth, ${{\rm{e}}}^{-z/H}$, where *z* is depth and where the scale height $H$ is a free parameter to be retrieved by matching the observed spectrum. These scatterers are assumed to follow a differential power-law size distribution $n\left(a\right)\propto {a}^{-\beta }.$ We fixed the minimum size ${a}_{min}=0.1\,{\rm{cm}}$, which is the smallest size that can be observed by MWR frequencies, and the maximum size ${a}_{max}=0.5\,{\rm{m}}$, noting that larger scatterers do not show a frequency dependence across any of the six MWR channels. The power-law index $\beta$ is a free parameter to be retrieved. The size of the scatterers determines the frequency dependence of the volume scattering. At the top surface, the reflection from pure solid ice to vacuum would be approximately 8%. However, non-icy materials on the surface or highly porous ice could increase or reduce the surface reflection. Thus, we characterized the microwave surface reflection as a free parameter *R*_S_.

The temperature profile within the diurnal layer is influenced by the local albedo, time of day and latitude and was characterized by solving the one-dimensional heat diffusion equation using a two-layer model. The thermal structure of the near-surface diurnal layer was calculated by solving the one-dimensional heat diffusion equation. We employed an exponentially increasing density profile, ranging from 200 kg m^−3^ at the surface to 934 kg m^−3^ at depths greater than 2 m. Because thermal conductivity depends on porosity and, thus, density, the thermal inertia also increases exponentially, rising from approximately 51 J m^−2^ K^−1^ s^−^^1/2^ at the surface to around 2,000 J m^−^^2^ K^−^^1^ s^−^^1/2^ at depth. The near-surface thermal inertia closely aligns with values reported in previous studies, which are constrained by observations from the photopolarimeter-radiometer onboard Galileo, whereas the deeper value is consistent with that of solid ice. (Note that this assumption was simply used in the calculation of the zero-order thermal structure, and we do not assume the absence of voids, fractures or other scatterers in the deep ice. Below, we use MWR data to constrain the scatterers.) The resulting temperature profile is a function of albedo, local solar time, latitude and depth. Beneath the diurnal layer, temperatures increase with depth following a lapse rate indicative of the heat flux, which was computed by following the description in ref. ^40^, based on a mixing length theory parameterization of convection that uses a temperature-dependent thermal conductivity^41^ and assumes no internal tidal heating. The temperature of the ice–water interface varies slightly with both pressure and salinity. We assumed pure water, which resulted in a temperature of 270 K for the ice–water interface in our best-fitting model. A salinity of 35 g kg^−1^ NaCl (the terrestrial value) would change this to 268 K, with a negligible impact on our analysis. The model shows that MWR measurements are insensitive to the viscosity and the possible existence of a deep convective layer, so our estimate of the conductive ice-shell thickness is a lower bound on the total ice-shell thickness. The presence of a convective ice layer would increase the total ice-shell thickness and reduce the temperature at the base of the conductive layer^18^, decreasing our estimate of the conductive ice-shell thickness by less than 2 km.

The dielectric constant of pure water ice has been quantified by Matzler et al.^27,42^. The reflected extrinsic radiation comprises three components: (1) reflection by the surface layer, (2) reflection by interior volume scatterers and (3) reflection by the bottom water–ice interface. Assuming that several reflections produce an approximately 30% reflection at the surface interfaces, as suggested for Ganymede by Brown et al.^25^, a perfectly smooth, mirror-like surface would result in a laterally varying increase in the brightness temperature of about 140 K at 0.6 GHz, with a maximum at approximately 10° N and 7° W, as shown in the middle panel of Supplementary Fig. 2. A smooth surface with a single reflection at the interface between vacuum and pure ice would result in about 8% reflection, scaling the brightness in Supplementary Fig. 2 by about a factor of 4. Comparing the observed brightness temperatures *T*_B_ at 0.6 GHz (Supplementary Fig. 2, left), there is no evidence of specular reflection (with a similar result at 1.2 GHz), indicating either a rough surface at 0.6 GHz/50 cm and 1.2 GHz/25 cm or the absence of surface reflection (for example, because of highly porous ice near the surface). The Lambertian-reflected JSR is proportional to the cosine of the incidence angle (Supplementary Fig. 2, right) and would show a correlation with the brightness at 0.6 GHz. The correlation coefficients between MWR brightness and the reflected JSR models are 0.67 for the specular case and 0.89 for the Lambertian case. When simulating the synchrotron radiation reflected by the surface layer, we treat the surface as Lambertian. In the alternative scenario, if surface reflection is nearly negligible, then JSR reflected by subsurface volume scatterers would also be proportional to the cosine of the incidence angle. We note that the scattering behaviour we observe is consistent with Goldstone and Arecibo radar observations of Europa at 2.3 GHz and 8.6 GHz (ref. ^43^).

We adapted the Discrete Ordinate Radiative Transfer (DISORT) code^44,45^ to our MWR Europa observations. The sounding depth of each channel was divided into 50 layers of ice. The required parameters for each layer *i* include physical temperature ${T}_{i}$, total optical depth ${\tau }_{i}$, single-scattering albedo ${\omega }_{i}$ and scattering phase function ${p}_{i}$. The total optical depth ${\tau }_{i}$ is composed of the optical depth for water-ice absorption ${\tau }_{i}^{{\rm{ice}}{\rm{\_}}{\rm{a}}}$ and the optical depth for volume-scattering extinction ${\tau }_{i}^{{\rm{v}}{\rm{\_}}{\rm{E}}}$, ${\tau }_{i}$ = ${\tau }_{i}^{{\rm{ice}}{\rm{\_}}{\rm{a}}}+{\tau }_{i}^{{\rm{v}}{\rm{\_}}{\rm{E}}}$. Here, ${\omega }_{i}={\tau }_{i}^{{\rm{v}}{\rm{\_}}{\rm{s}}}/({\tau }_{i}^{{\rm{ice}}{\rm{\_}}{\rm{a}}}+{\tau }_{i}^{{\rm{v}}{\rm{\_}}{\rm{E}}})$, where ${\tau }_{i}^{{\rm{v}}{\rm{\_}}{\rm{s}}}$ is the optical depth for volume scattering. For scatterers with volume fraction ${f}_{i}$, ${\tau }_{i}^{\mathrm{ice\_a}}$ $=\frac{4{\rm{\pi }}{n}_{i}({T}_{i})}{\nu }\,{{\rm{d}}z}_{i}\,(1-{f}_{i})$, where ${{\rm{d}}z}_{i}$ is the thickness of layer *i*. ${n}_{i}({T}_{i})$ is the imaginary part of the water-ice refractive index at physical temperature ${T}_{i}$, which was computed using the Maztler model. For scatterers with power-law index $\beta$, the extinction coefficient ${Q}_{{\rm{ext}}}^{i}$, scattering coefficient ${Q}_{{\rm{sca}}}^{i}$ and scattering phase function ${p}_{i}$ were computed using Mie theory. Pores were assumed to be spherical or randomly oriented. However, DISORT does not account for reflection at the dielectric interface when upwelling radiation reaches the surface. To approximate this process, we tracked the radiation that is reflected back and treated it as an incident beam into the ice shell. This process was repeated three times to ensure that the remaining radiation correction was negligible.

The reflected JSR and GR are each composed of a surface reflected part and a volume-scatterer reflected part. The surface reflected part was integrated over solid angle $$\varOmega$$ as $${\int }_{0}^{2{\rm{\pi }}}{\int }_{0}^{{\rm{\pi }}/2}{T}_{{\rm{B}},\mathrm{JSR}/\mathrm{GR}}\cos {\theta }_{\mathrm{incid}}\frac{{R}_{{\rm{s}}}}{{\rm{\pi }}}\,{\rm{d}}\varOmega$$, with incidence angle *θ*_incid_, where *T*_B,JSR/GR_ is the brightness temperature of the JSR and GR components. The volume-scatterer reflected part was computed with the DISORT code with incident JSR and GR flux with further treatment for the transmission between the vacuum and solid ice. For incident flux in the vacuum, $${F}_{0}={\int }_{0}^{2{\rm{\pi }}}{\int }_{0}^{{\rm{\pi }}/2}{\varepsilon }_{\mathrm{vacuum}}{T}_{\mathrm{B,JSR/GR}}\,{\rm{d}}\varOmega$$, the incident flux in the solid ice medium after transmission through the vacuum–ice interface becomes $${F}_{1}={F}_{0}(1-{R}_{{\rm{s}}})\cos {\theta }_{\mathrm{incid}}/\cos {\theta }_{\mathrm{incid}}^{{\prime} }$$, where $$\sqrt{{\varepsilon }_{\mathrm{vacuum}}}\sin {\theta }_{\mathrm{incid}}=$$$$\sqrt{{\varepsilon }_{\mathrm{ice}}}\sin {\theta }_{\mathrm{incid}}^{{\prime} }$$, where *ε*_vacuum_ is the vacuum dielectric constant and *ε*_ice_ is the ice dielectric constant. The DISORT code computes the distribution of intensity $${I}_{2}(\theta ,\varphi )$$ as a function of zenith angle *θ* and azimuthal angle $$\varphi$$ in the ice medium near the surface before reaching the ice–vacuum interface. After transmission through the ice–vacuum interface, the distribution of intensity in the vacuum $${I}_{3}({\theta }^{{\prime} },{\varphi }^{{\prime} })=\frac{{I}_{2}(\theta ,\varphi ){\varepsilon }_{\mathrm{vacuum}}}{{\varepsilon }_{\mathrm{ice}}}(1-{R}_{{\rm{s}}})$$, where $${\varepsilon }_{{\rm{vacuum}}}=1$$ and $${\varepsilon }_{{\rm{ice}}}=3.03$$ at 100 K for 0.6 GHz and 1.2 GHz.

For a globally uniform model, the free parameters to be retrieved included surface reflection ${R}_{s}$, the volume fraction of volume scatterers near the top surface ${f}_{v}$, the exponentially decreasing scale height $H$ and the power-law index for the volume-scatterer size distribution $\beta$. We employed an MCMC retrieval process to estimate the free parameters. We matched the model to the observed brightness temperature at all six frequencies and at all locations simultaneously. We used the Python PyMC3 module^29^, which provides robust and efficient sampling methods for Bayesian inference. In addition to the model parameters above, we fitted an instrumental offset *σ*_n_ in the measurement of *T*_B_ for each channel, constrained to within 0.5 K, 0.7 K, 1.0 K, 0.3 K, 0.4 K or 0.2 K of zero^26^, and we used 1.3 K, 1.9 K, 3.0 K, 3.5 K, 3.4 K and 3.2 K for the 0.6–22-GHz channels, respectively, as the data uncertainties for the likelihood function.

As shown in Figs. 2–4, the data are fitted best by a model with a conductive ice-shell thickness of 29 km. The parameters retrieved for this 29-km conductive ice-shell model are ${R}_{{\rm{s}}}$ = 0.004, ${f}_{v}$ = 0.045, $H$ = 219 m and $\beta$ = 3.96. See Supplementary Fig. 3. Supplementary Figs. 4–7 show examples with conductive ice-shell thicknesses of 20 km and 40 km, respectively. The parameters retrieved for an ice-shell thickness of 20 km are ${R}_{s}$ = 0.002, ${f}_{v}$ = 0.041, $H$ = 586 m and $\beta$ = 4.19, whereas the parameters retrieved for an ice-shell thickness of 40 km are ${R}_{s}$ = 0.017, ${f}_{v}$ = 0.025, $H$ = 289 m and $\beta$ = 3.95. A thinner (thicker) ice shell results in a steeper (shallower) vertical temperature gradient, requiring more (less) reflection at 0.6 GHz to match the observations. The angular dependence of the illumination of the icy surface by the JSR produces horizontal gradients in the reflected emission at 0.6 GHz, which provides another constraint on the reflectivity. The mismatch between the model and the data at 0.6 GHz for the models shown in Supplementary Figs. 4–7 results from a conflict between these two constraints.

### Lateral variation

As shown in Fig. 3, a large part of the lateral variation in *T*_B_ is probably caused by variations in the ice temperature. To examine the other causes of lateral variation, we subtracted the model shown in Fig. 3 and examined the residual. As shown in Supplementary Fig. 8, the remaining component of the lateral variation in the three shallowest channels is highly correlated, with an amplitude that increases with depth. This can be explained by a lateral variation in the subsurface reflectors, with a depth that extends below the sounding depth at 10 GHz. Similarly, subtracting the linear fit shown in Supplementary Fig. 8 shows a smaller residual of the lateral variation caused by scatterers that extend below the sounding depth of the 5.2-GHz channel, as shown in Supplementary Fig. 9. We continue the process with Supplementary Fig. 10, finding a small residual in the variation due to scatterers that extend below the sounding depth of the 2.5-GHz channel. That this lateral variation is largely absent from the deepest channels may well indicate that the lateral variation is restricted to the first few hundred metres, but the size of the scatterers may also play a role.

## Supplementary information

Supplementary Information Supplementary Figs. 1–10, including captions.

## Data Availability

Juno MWR data are archived in the NASA Planetary Data System on the Planetary Atmospheres Node at https://pds-atmospheres.nmsu.edu/data_and_services/atmospheres_data/JUNO/microwave.html.
