# Supplementary material for: Europa’s ice thickness and subsurface structure characterized by the Juno microwave radiometer
Source: Nat Astron. 2025 Dec 17;10(1):84–91. doi: 10.1038/s41550-025-02718-0 (PMC12827049; doi:10.1038/s41550-025-02718-0)
Supplement: Supplementary file 1 — Supplementary Figs. 1–10, including captions. [file 41550_2025_2718_MOESM1_ESM.pdf]

nature astronomy

Supplementary information https://doi.org/10.1038/s41550-025-02718-0

Europa's ice thickness and subsurface structure characterized by the Juno microwave radiometer

In the format provided by the authors and unedited

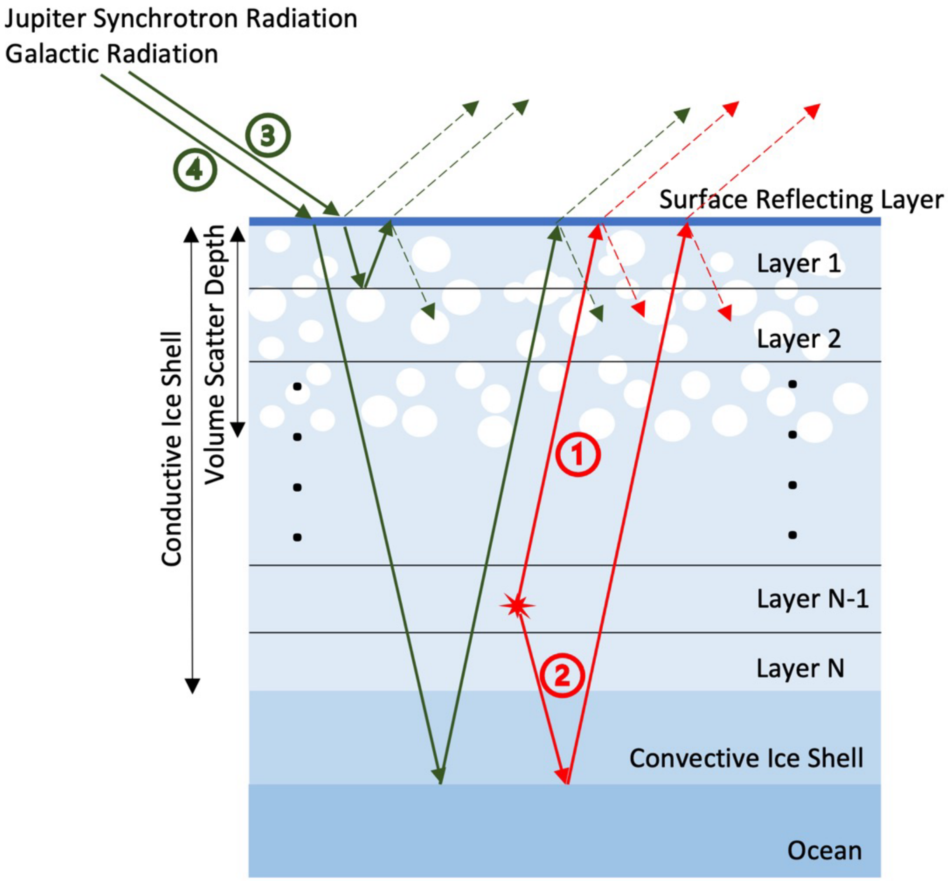

Supplementary Figure 1. Schematic structure of the radiative transfer model. Our model incorporates a reflection at the surface and volume scattering in the subsurface. Volume scattering is assumed to be caused by voids in the medium of solid ice.

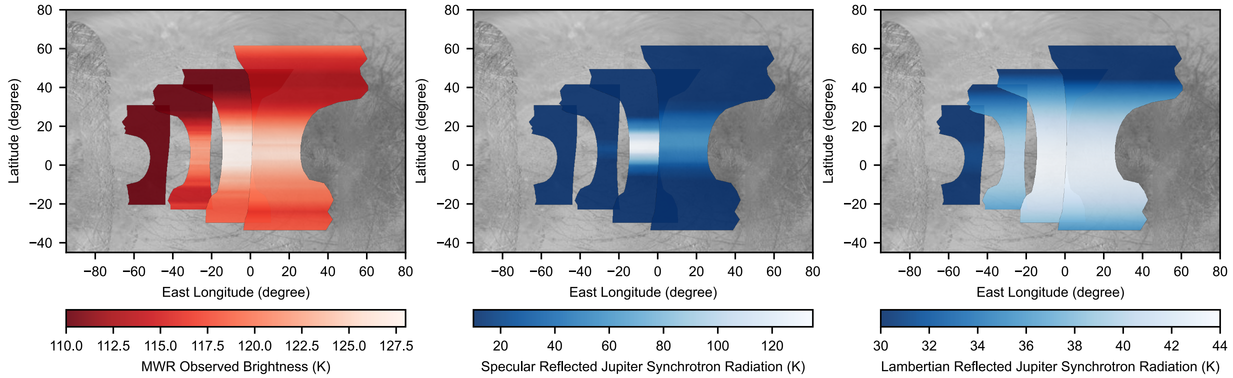

Supplementary Figure 2. Maps of MWR observed brightness temperature (left), specular reflected Jovian synchrotron radiation (JSR) (middle) and Lambertian reflected Jupiter synchrotron (right) for 0.6 GHz. The specular reflected Jupiter synchrotron is computed by convolving with the microwave radiometer experiment (MWR) beam pattern, assuming a perfectly smooth surface and microwave reflectivity of 30%. The specular reflected Jupiter synchrotron would have the largest contribution (~140K at ~10°N/7°W) if present. The relatively small variation (<<70K) at 0.6 GHz suggests a non-smooth surface. The sub-Jovian point is at 0° latitude and longitude. The Lambertian reflected Jupiter Synchrotron is proportional to the cosine of the incidence angle. The width of each swath indicates the half-power beamwidth as projected onto the surface.

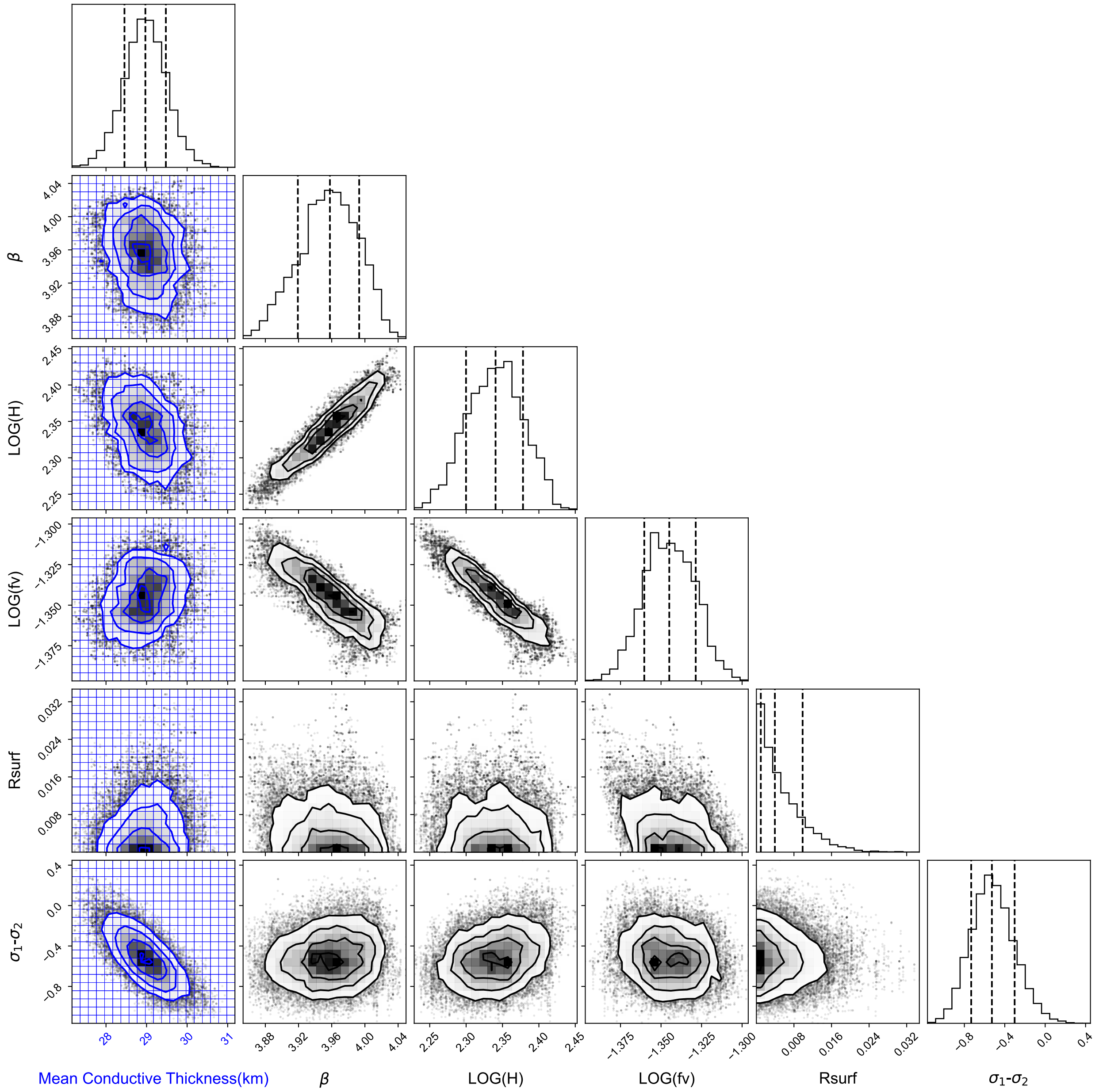

Supplementary Figure 3. Distribution of select parameters from MCMC modeling.

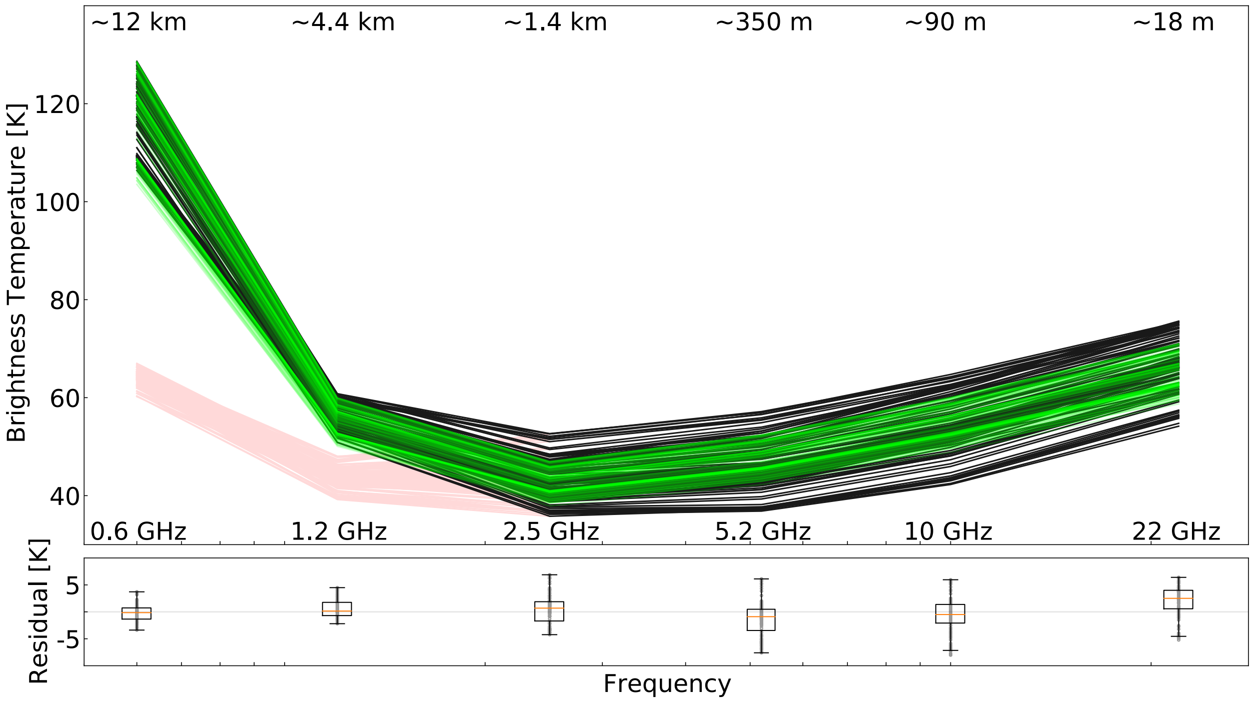

Supplementary Figure 4. Microwave data (black) and modeled (light green) spectra from Europa, with the conductive ice shell thickness constrained to 20 km. Pink spectra represent data after subtracting modeled effects of reflected Jovian synchrotron and galactic emission. Approxiate depth in pure, solid, ice for 50% contribution to each channel is shown at the top. Residuals for the 129 measurements (data minus model) are shown at the bottom, with boxes indicating median, first and third quartiles, and 1.5 times the inter-quartile range.

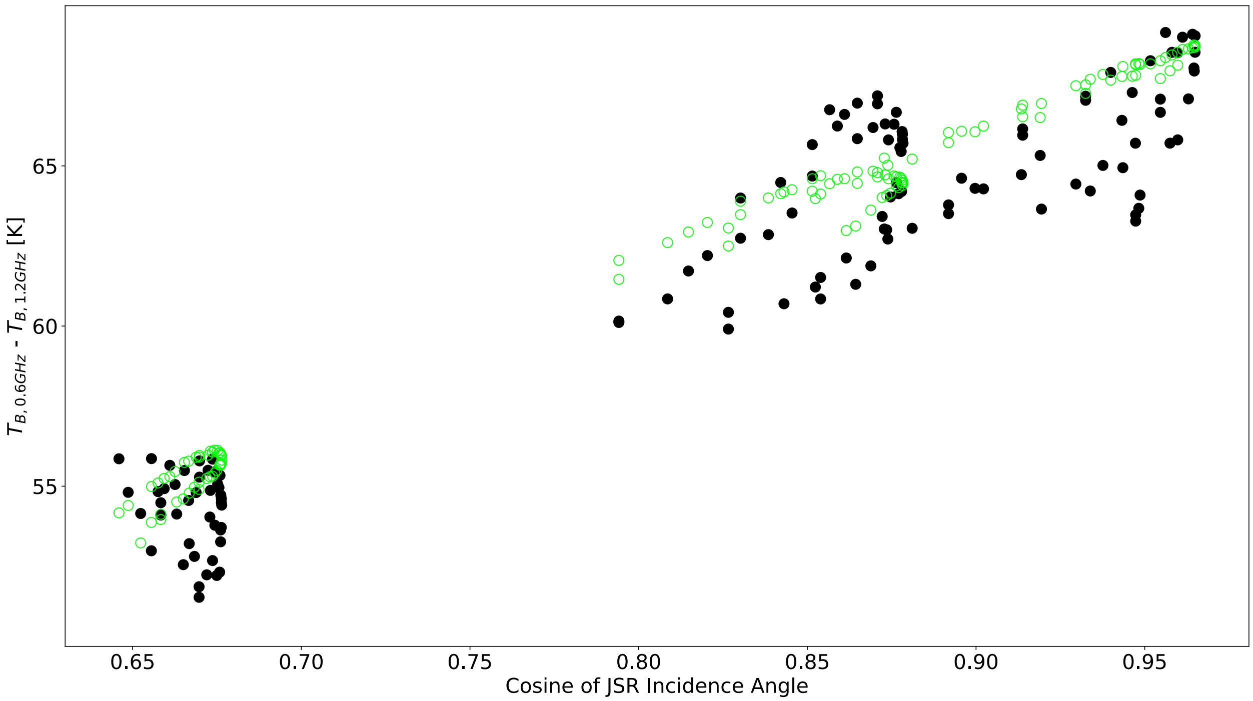

Supplementary Figure 5. Brightness temperature gradient between 0.6 Ghz and 1.2 GHz, observed (black filled circles) and modeled (light green open circles), plotted against cosine of the incidence angle for synchrotron emission. The model is the same as in Supplementary Figure 4.

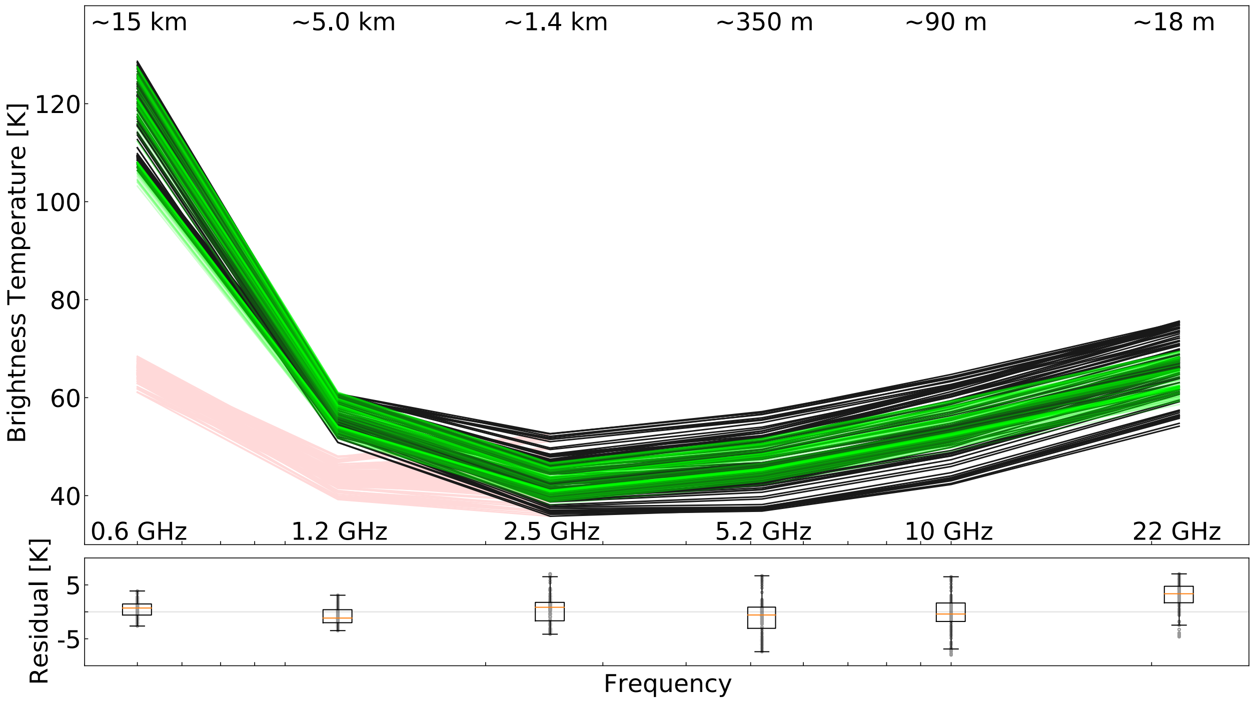

Supplementary Figure 6. Microwave data (black) and modeled (light green) spectra from Europa, with the conductive ice shell thickness constrained to 40 km. Pink spectra represent data after subtracting modeled effects of reflected Jovian synchrotron and galactic emission. Approxiate depth in pure, solid, ice for 50% contribution to each channel is shown at the top. Residuals for the 129 measurements (data minus model) are shown at the bottom, with boxes indicating median, first and third quartiles, and 1.5 times the inter-quartile range.

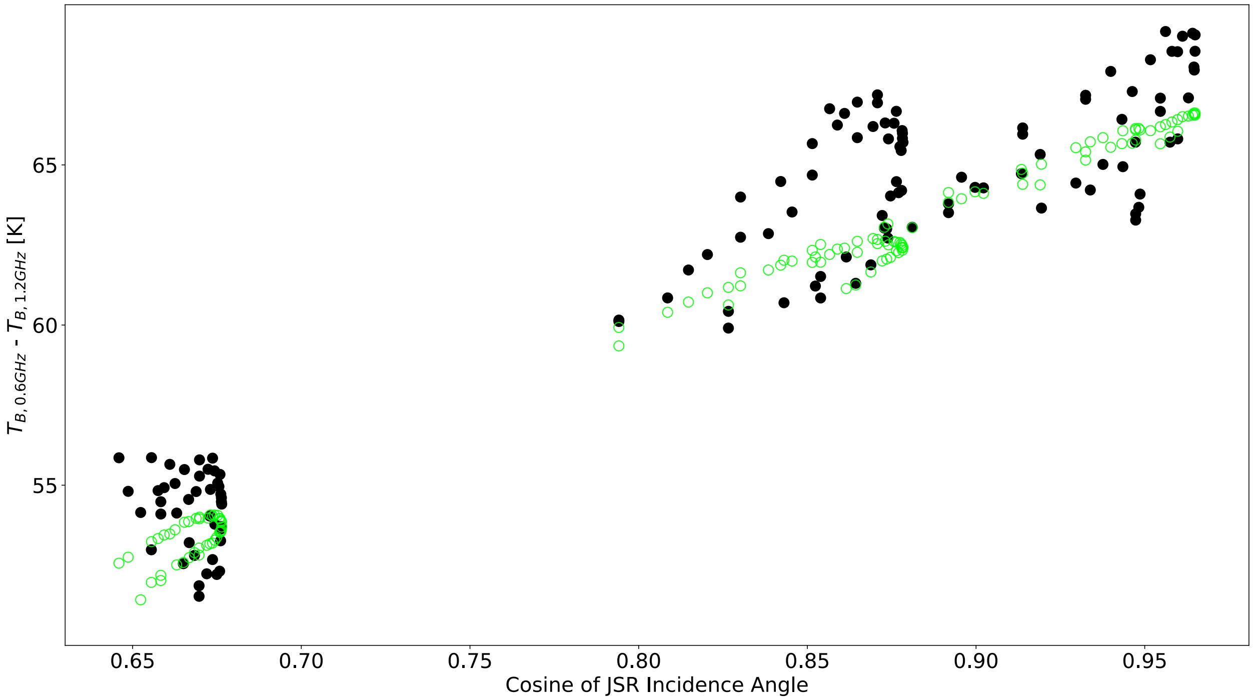

Supplementary Figure 7. Brightness temperature gradient between 0.6 Ghz and 1.2 GHz, observed (black filled circles) and modeled (green open circles), plotted against cosine of the incidence angle for synchrotron emission. The model is the same as in Supplementary Figure 6.

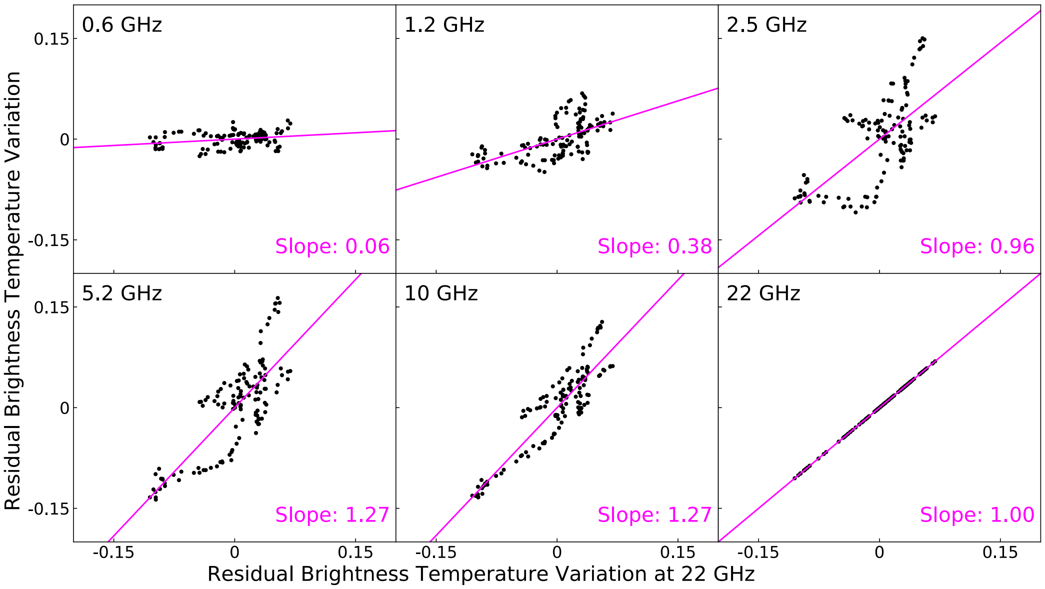

Supplementary Figure 8. Variation in brightness temperature after removing the model shown in Figure 3, plotted vs the corresponding variation in the 22 GHz channel.

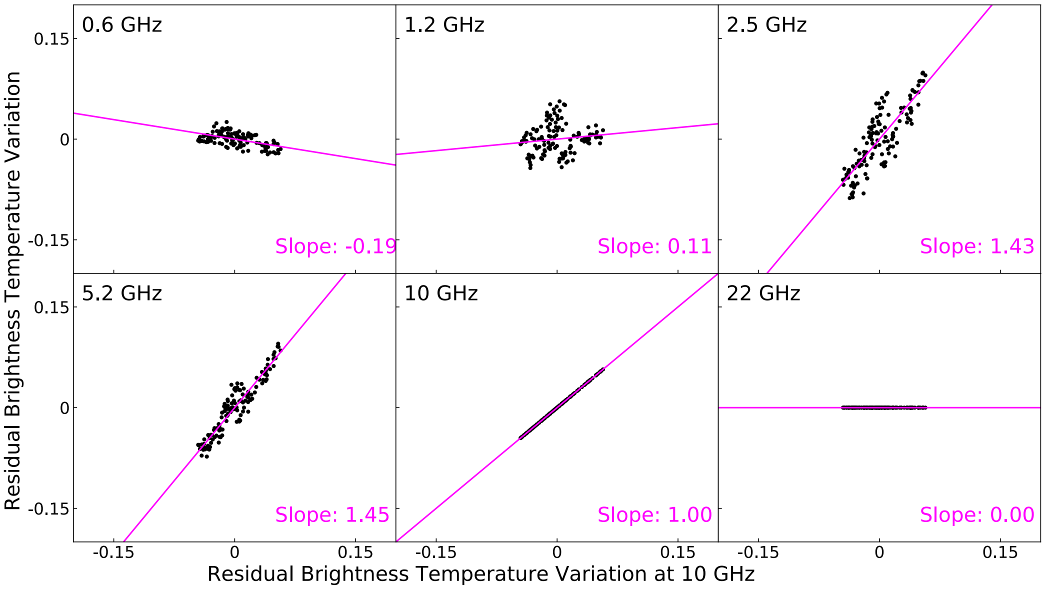

Supplementary Figure 9. Variation in brightness temperature after removing the variation shown in Supplementary Figure 8, plotted vs the corresponding variation in the 10 GHz channel.

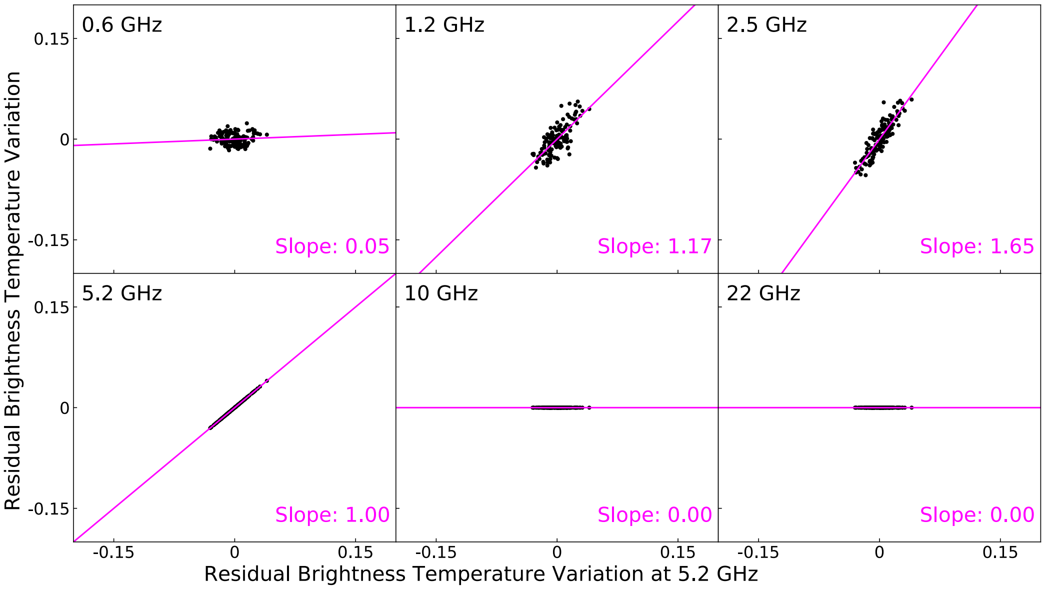

Supplementary Figure 10. Variation in brightness temperature after removing the variation shown in Supplementary Figure 9, plotted vs the corresponding variation in the 5.2 GHz channel.
